# Reducing the incidence of stroke-associated pneumonia: an evidence-based practice

**DOI:** 10.1186/s12883-022-02826-8

**Published:** 2022-08-11

**Authors:** Zhu-Yun Liu, Lin Wei, Ri-Chun Ye, Jiao Chen, Dan Nie, Ge Zhang, Xiao-Pei Zhang

**Affiliations:** 1 Department of Neurology, The Second Affiliated Hospital of Guangzhou University of Chinese Medicine, No.111, Dade Road, Yuexiu District, Guangzhou, China; 2 Nursing department, The Second Affiliated Hospital of Guangzhou University of Chinese Medicine, No.111, Dade Road, Yuexiu District, Guangzhou, China; 3 Development Research Center of Chinese Medicine, The Second Affiliated Hospital of Guangzhou University of Chinese Medicine, No.111, Dade Road, Yuexiu District, Guangzhou, China

**Keywords:** Stroke, Pneumonia, Evidence-based practice, Quality improvement, Prevention, Patient safety

## Abstract

**Background:**

Pulmonary infection is a frequent complication among stroke patients and adversely affects clinical outcomes, increases the length of hospitalization stay and costs, and aggravates the financial burden of the national medical health system. Early identification and management of high-risk patients are necessary and imperative to reduce the incidence of stroke-associated pneumonia (SAP).

**Aim:**

The evidence-based practice project evaluated the effectiveness of a standard care bundle intervention in preventing the occurrence of SAP.

**Methods:**

The project was conducted in a neurology department of a teaching hospital. Given the variation in assessment and management standards, evidence-based practice (EBP) methodology was used to establish a process for quality improvement. A thorough literature search was conducted to identify evidence-based interventions to manage and prevent SAP. Thorough critiques of the literature and synthesis of the evidence were completed. A systematic management flow and care bundle interventions were established. The care bundle included interventions, such as the utilization of tools for SAP risk screening; dysphagia screening and rehabilitation; feeding modification, oral care, airway management, position management, and the nursing techniques of traditional Chinese medicine.

**Results:**

A significant improvement was observed in preventing SAP in patients in the postimplementation group compared with those in the preimplementation group (14.0% vs. 37.2%, *p* = 0.025). In addition, significantly lower duration of hospitalization, lower rate of aspiration, and improvements in albumin and oral hygiene were found after the implementation of the care bundle.

**Conclusions:**

Evidence-based care bundles successfully empower nurses to reduce the incidence of SAP. The management flow of SAP prevention could be promoted to other units of the neurology department in the future. The results of the project reflect positively on the capacity to implement EBP in an acute care setting for stroke. The EBP methodology can be utilized to solve other clinical problems.

**Supplementary Information:**

The online version contains supplementary material available at 10.1186/s12883-022-02826-8.

## Introduction

Stroke is a widely prevalent acute cerebrovascular disease with high morbidity and mortality [1]. Acute ischaemic stroke accounts for 60–80% of all strokes [1, 2]. Pulmonary infections frequently occur among stroke patients and adversely affect clinical outcomes, aggravating the financial burden on family and national medical health systems [3–5].

Stroke-associated pneumonia (SAP) refers to pulmonary infections that develop within the first 7 days of stroke onset among nonventilated patients, affecting 2.3 to 44% of stroke patients [6, 7]. Female sex, advanced age, dysphagia, the severity of acute stroke and disturbance of consciousness are the main risk factors for SAP [8–11]. Some of these factors have been used as scoring items in the scale to assess the risk of SAP. The combination of aspiration caused by these risk factors and acute stroke-induced immunosuppression leads to the increased incidence of SAP [12, 13].

Previous studies have shown that SAP can be prevented [14, 15]. In addition, 43–79% of SAP occurs within 72 h of acute stroke onset [14–16]. Therefore, early identification and management of high-risk patients are necessary to reduce the incidence of SAP [17].

Although many scholars have explored and evaluated the effects of different preventive interventions on SAP from different perspectives, systematic prevention recommendations or management flowcharts for SAP have not yet been developed. Effective nursing interventions include feeding management, respiratory tract management, dysphagia rehabilitation, cluster nursing interventions, and traditional Chinese medicine (TCM) nursing [9, 18, 19]. It is necessary to develop an evidence-based nursing scheme based on extensive evidence, including TCM nursing interventions.

## Methods

### Context

This project was implemented in the Neurology Department of The Second Affiliated Hospital of Guangzhou University of Traditional Chinese Medicine, which is a cerebrovascular disease medical center with more than 300 beds. A 28-bed internal medicine unit with more than 1200 annual cerebrovascular disease visits was designated for the project. The prevalence rate of SAP in the unit was 26.5% in 2018.

### Purpose

This nurse-led project aimed to institute a best practice bundle and management process to reinforce systematic management of patients with acute stroke, resulting in a decrease in the incidence of SAP.

### Population

The population involved in the project was a convenience sample from September 2020 to May 2021. The inclusion criteria were as follows: (1) diagnosed with ischaemic stroke by computed tomography (CT) or magnetic resonance imaging (MRI); (2) between 18 and 80 years old; (3) time from symptom onset within 7 days; (4) admission without pulmonary infection; and (5) A^2^DS^2^ (Age, Atrial fibrillation, Dysphagia, Sex, Stroke Severity) score between 5 and 10. The exclusion criteria were: (1) a history of mental diseases; and (2) a history of severe diseases of the heart, liver and kidney (including malignant tumor of heart, liver, and kidney, acute myocardial infarction, allogeneic transplantation of the heart, liver, and kidney, end-stage renal disease or chronic renal failure and uremia, acute or subacute severe hepatitis, decompensated chronic liver failure, severe primary cardiomyopathy).

Sample size estimation was based on a similar study, which adopted an evidence-based care bundle to decrease the incidence of SAP from 43.3 to 16.7% [19]. With a power of 0.80, an alpha set at 0.05, and an effect size for the primary outcome (rate of SAP), each group required 43 participants.

### Project design: the Iowa model

The Iowa Model of Evidence-Based Practice was utilized as a framework for project execution [20]. SAP in acute stroke patients was a problem-focused trigger, which was of high priority to the neurology unit. An interdisciplinary team was formed and responsible for the development and implementation of the evidence-based practice change. Relevant publications were gathered and evaluated for reliability, validity, and bias. The researchers found sufficient studies with consistent findings to support the following practice change.

The clinical nurse specialist conducted a systematic literature search. The goal was to identify what evidence-based interventions might exist to decrease the incidence of SAP. The Population, Intervention, Comparison, Outcome (PICO) framework was used to develop the search strategy: in patients with acute stroke, how do evidence-based nursing bundle interventions compared to conventional nursing interventions affect SAP rates? A Boolean search was completed using the following terms: (“acute stroke” OR “ischaemic acute stroke” OR “cerebral infraction” OR “cerebrovascular accident” OR “cerebrovascular apoplexy”) AND (“pneumonia” OR “stroke associated pneumonia” OR “SAP” OR “aspiration pneumonia” OR “inhalation pneumonia” OR “lung inflammation” OR “labor pneumonia”). The databases searched included the National Institute for Clinical Excellence (NICE), National Guideline Clearinghouse (NGC), Scottish Intercollegiate Guidelines Network (SING), MEDLINE, PubMed, Embase, Cochrane Library, Joanna Briggs Institute (JBI), EBSCO, Web of Science, Ovid, Chinese National Knowledge Infrastructure (CNKI), Wan Fang Data, VIP, and SinoMed. The inclusion criteria allowed articles about randomized controlled trials, quasi-experimental studies, case-control studies, and cohort studies published between 2009 and 2018. The search yielded 4766 articles and included 16 articles after critical appraisal with tools of Joanna Briggs Institute and AGREE II. These included 4 evidence-based clinical guidelines from America, England and Canada, 3 Chinese clinical guidelines and expert consensus, 3 evidence synthesis, 1 systematic review, 2 cohort trails and 3 random controlled trials. Evidence-based practice interventions were based on high quality clinical evidence and emphasized clinical expertise, patient values and expectations [21]. A gap analysis of current practice was completed to identify opportunities for improvement. Evidence-based knowledge was then applied to clinical practice through staff education and training, equipment availability, and environmental adjustments. The management scheme and flow were revised and detailed by consulting clinical medical, rehabilitation and nurse specialists and staff, who provided final feedback and adjustments based on FAME (feasibility, appropriateness, meaningfulness, effectiveness). Before implementing the final interventions, meetings were held to educate other providers and support staff (medical assistants and schedulers) about the project goals and to ask for their feedback. A 2-week period pilot study was conducted to assess feasibility and any improvement in outcomes.

### Practice changes

The study was approved by the ethical committee of The Second Affiliated Hospital of Guangzhou University of Traditional Chinese Medicine. Based on a literature review, SAP management recommendations included dysphagia screening and management, feeding management, oral hygiene management, position management, and TCM nursing techniques (Figs. 2 and Supplement 1).

### Nurses’ preparation before practice changes

Multiple staff meetings and face-to-face discussions were organized, and electronic materials were provided through the WeChat app to educate nursing staff about the bundle intervention protocol. Nursing education was done during a 2-week period before implementing the bundle nursing practice. The whole training was completed by the first author, and swallowing screening and rehabilitation were trained by senior speech and language therapists specializing in neurological rehabilitation. During the training process, the SAP prevention and management manual was produced based on the evidence, and sought for the support of the department head and rehabilitation therapists. At the end of the study, the nurses received a comprehensive real-situation examination by one senior speech therapist and one head nurse of the unit that included dysphagia screening, formulation and implementation of rehabilitation recommendations, and patient education. Those who passed the examination could implement the evidence-based care bundle in the stroke unit.

### Data collection and analyses

The impact of the evidence-based care bundle on the rate of SAP and related patient outcomes was evaluated using data collected directly from patients and electronic medical records. In this study, SAP was diagnosed based on the medical documents and re-evaluated by the treating physician according to American CDC’s (Centers for Disease Control and Prevention) diagnostic criteria [22] for pneumonia (Supplement 2). This diagnostic standard is also recommended by the Consensus of Chinese experts on the diagnosis and treatment of stroke-associated pneumonia in 2019.

**Fig. 1:**
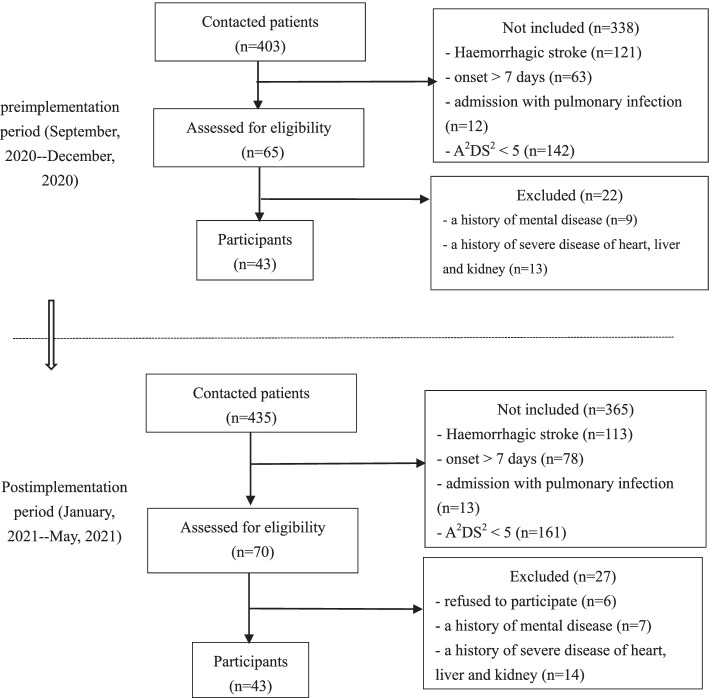
The flowchart of the study. A^2^DS^2^: Age, Atrial fibrillation, Dysphagia, Sex, Stroke Severity

Patient-related data collected after implementation of the evidence-based care bundle (January 2021 to May 2021, prospective) were compared with data from the preimplementation period (September 2020 to December 2020, retrospective) (Fig. 1). Baseline data collected at admission included: age; sex; education; location of acute stroke; smoking; alcohol consumption; complications (diabetes, hypertension, heart disease); A^2^DS^2^ score; the results of 30 ml water swallowing test; aspiration (Supplement 3); National Institutes of Health Stroke Scale (NIHSS); oral hygiene [23]; nutrition risk screening-2002 (NRS-2002); serum albumin (ALB); white blood cell (WBC); procalcitonin (PCT); and hypersensitive C-reactive protein (CRPH). Data collected on the 7th day of admission were NIHSS, NRS-2002, ALB, WBC, PCT, CRPH, and incidence of SAP. The length of hospital stay and medical cost were collected at discharge. All data were collected and stored by the advanced practice nurse.

**Fig. 2:**
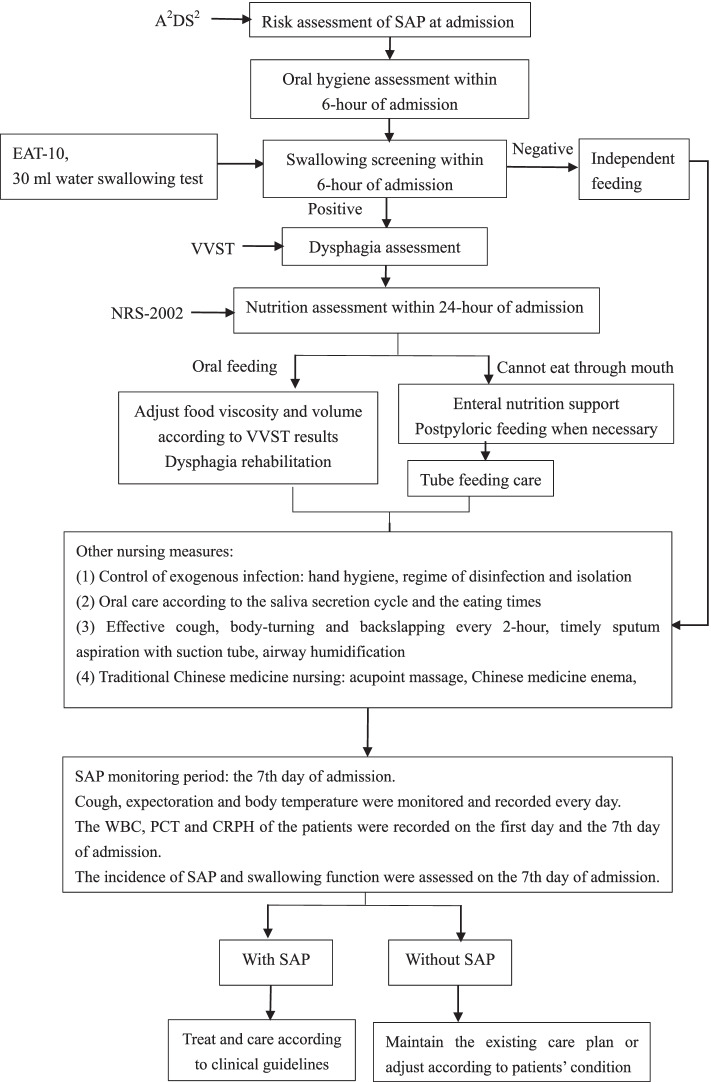
The workflow of SAP prevention of patients with stroke

Data analysis included descriptive statistics for all variables. The differences between the preimplementation and postimplementation groups were compared by the χ^2^ test, Fisher’s exact test, and Student’s t test or Mann–Whitney U test as appropriate. All data analysis was completed in SPSS version 22.0 for Windows (SPSS Inc., Chicago, IL, USA).

## Results

### Patients’ characteristics

Table 1 presents the participants’ demographic, neurological and related data. There were no significant differences between the two groups in their baseline characteristics. There were also no significant differences between the two groups in infection-related indicators of SAP, which included WBC, PCT and CRPH (Table 2).

**Table 1:** Baseline characteristics of the study population (*n* = 86)

Variables	Preimplementation group (*n* = 43)	Postimplementation group (n = 43)	*Z/F/U/χ*^*2*^	*P*
Age, years old	69 (64, 76)	68 (63, 74)	846.0	0.497
Sex, males	24 (55.8)	23 (53.5)	0.047	0.829
Employment			2.708	0.305^a^
Employed	1 (2.4)	5 (11.6)		
Retired	35 (85.4)	32 (74.4)		
Unemployed	5 (12.2)	6 (14.0)		
Risk factors				
Hypertension	33 (76.7)	31 (72.1)	0.244	0.805
Diabetes	13 (30.2)	15 (34.9)	0.212	0.818
Dyslipidemia	10 (23.3)	12 (27.9)	0.244	0.805
Atrial fibrillation	14 (32.6)	7 (16.3)	3.087	0.131
Smoking	11 (25.6)	14 (32.6)	0.508	0.635
Alcohol consumption	4 (9.3)	8 (18.6)	1.550	0.351
Stroke etiology			2.437	0.543^a^
Cardioembolism	12.3)	0 (0.0)		
Large-vessel disease	24 (55.8)	30 (69.8)		
Small-vessel disease	15 (34.9)	11 (25.6)		
Other causes	3 (7.0)	2 (4.7)		
OCSP classification			0.497	0.946
Total anterior circulation infract	7 (16.3)	7 (16.3)		
Partial anterior circulation infract	24 (55.8)	25 (58.1)		
Posterior circulation	6 (14.0)	7 (16.3)		
Lacunar infarct	6 (14.0)	4 (9.3)		
Treatment protocols				0.483^a^
Drugs	37 (86.0)	40 (93.0)		
Non-drugs	6 (14.0)	3 (7.0)		
Admission NIHSS score	6 (3, 13)	6 (4, 10)	879.0	0.693
Admission BP, mmHg				
Systolic BP	160 (140, 175)	153 (136, 175)	842.0	0.476
Diastolic BP	87 (76, 95)	91 (81, 96)	788.5	0.240
A^2^DS^2^ score	6 (5, 6)	6 (5, 7)	802.5	0.262
Oral hygiene score	23 (17, 26)	22 (18, 26)	903.5	0.856
NRS-2002			0.05	1.000
≥ 3	27 (62.8)	28 (65.1)		
< 3	16 (37.2)	15 (34.9)		
30 ml water swallowing test			2.828	0.243
III	22 (51.2)	23 (53.5)		
IV	13 (30.2)	17 (39.5)		
V	8 (18.6)	3 (7.0)		
The degree of dysphagia			2.729	0.395^a^
3	1 (2.3)	0 (0.0)		
4	28 (65.1)	23 (53.5)		
5	12 (27.9)	18 (41.9)		
6	2 (4.7)	2 (4.7)		

^*^The difference was statistically significant

^a^Fisher’s exact test

Results are presented as n (%), mean ± standard deviation or median (interquartile range)

*OCSP* Oxfordshire Community Stroke Project; *NIHSS* National Institutes of Health Stroke Scale; *BP* blood pressure; *A*^*2*^*DS*^*2*^ Age, Atrial fibrillation, Dysphagia, Sex, Stroke Severity; *NRS* nutrition risk screening

**Table 2:** Outcome indicators of the study population on the day of admission (n = 86)

Variables	Preimplementation group (n = 43)	Postimplementation group (n = 43)	*F/U /χ*^*2*^	*P*
Aspiration	0 (0.0)	0 (0.0)		/
White blood cells (10^9^/L)	7.43 (5.89, 8.93)	7.30 (5.60, 8.30)	784.5	0.226
Procalcitonin (ng/ml)	0.045 (0.033, 0.056)	0.048 (0.036, 0.072)	737.5	0.106
Hypersensitive C-reactive protein (mg/L)	5.20 (4.02, 6.92)	5.10 (3.24, 6.81)	819.0	0.362
Hemoglobin (g/L)	116 (108, 136)	120 (109, 133)	865.0	0.607
Serum albumin (g/L)	36.93 ± 3.29	38.24 ± 3.31	−1.838	0.070

^a^Fisher’s exact test

Results are presented as n (%), mean ± standard deviation or median (interquartile range)

### SAP

After the evidence-based process was implemented for 6 months, outcome evaluations were completed. Six of 43 (14.0%) patients in the postimplementation group were identified as diagnosed SAP, which was significantly lower than that in the preimplementation group (14.0% vs. 37.2%, *p* = 0.025). After the evidence-based care bundle implementation, the postimplementation group had lower levels of PCT (*p* = 0.007) and CRPH (*p* = 0.013) than the preimplementation group (Table 3).

**Table 3:** All indicators of the study population (n = 86)

Variables	Preimplementation group (n = 43)	Postimplementation group (*n* = 43)	*t/U /χ*^*2*^	*P*
SAP	16 (37.2)	6 (14.0)	6.108	0.025^*^
Indicators of infection				
White blood cells (10^9^/L)	7.49 (5.51, 9.30)	7.20 (5.51, 8.40)	863.5	0.598
Procalcitonin (ng/ml)	0.083 (0.054, 0.095)	0.054 (0.044, 0.076)	610.5	0.007^*^
Hypersensitive C-reactive protein (mg/L)	8.74 (4.56, 13.60)	5.20 (3.40, 6.70)	636.5	0.013^*^
Length of stay, days	13 (9, 18)	11 (8, 14)	687.0	0.040^*^
Total cost, USD	20,351.62 (12,794.51, 32,790.98)	18,139.21 (12,658.4, 32,666.31)	843.0	0.481
NIHSS score	4 (2, 13)	6 (2, 9)	923.0	0.990
Aspiration	16 (37.2)	4 (9.3)	9.382	0.004^*^
NRS-2002			2.549	0.171
≥ 3	32 (74.4)	25 (58.1)		
< 3	11 (25.6)	18 (41.9)		
Serum albumin (g/L)	33.92 ± 2.90	35.83 ± 4.20	−2.459	0.016^*^
Oral hygiene score	19 (15, 21)	15 (13, 18)	530.5	0.001^*^
30 ml water swallowing test			6.766	0.149
I	2 (4.7)	10 (23.3)		
II	15 (34.9)	12 (27.9)		
III	16 (37.2)	15 (34.9)		
IV	6 (14.0)	4 (9.3)		
V	4 (9.3)	2 (4.7)		
The degree of dysphagia			7.097	0.131
0	2 (4.7)	10 (23.3)		
2	15 (34.9)	12 (27.9)		
4	17 (39.5)	16 (37.2)		
5	6 (14.0)	4 (9.3)		
6	3 (7.0)	1 (2.3)		

^*^The difference was statistically significant

Results are presented as n (%), mean ± standard deviation or median (interquartile range)

*SAP* stroke-associated pneumonia; *NIHSS* National Institutes of Health Stroke Scale; *NRS* nutrition risk screening

### Other findings

The mean length of hospital stay decreased from 14.47 ± 6.68 days before implementation to 11.74 ± 5.51 days after implementation. The cost of medical care of the postimplementation group was lower than that of the preimplementation group, but the difference was not statistically significant. The rate of aspiration of the postimplementation group was significantly lower than that of the preimplementation group. In addition, there were significant improvements in ALB, and oral hygiene (Table 3). The nurses’ compliance with using the entire care bundle was 77%.

## Discussion

This project instituted an evidence-based practice bundle in the management of acute stroke patients to successfully reduce the incidence of SAP. The focus on dysphagia screening and rehabilitation; feeding modification, oral care, airway management, position management, and TCM nursing techniques were strategies introduced through the care bundle.

SAP can be prevented in a variety of ways. Many other important factors, such as head position, getting out of bed, continuous tube feeding, caregivers’ feeding experience, aspiration caused by vomiting or gastroesophageal reflux, oral hygiene, immunosuppression after acute stroke, and the lesion location of dysphagia, are also linked to the occurrence of SAP and must be considered in care plans to prevent respiratory infections in patients with acute stroke [24, 25]. Many previous studies have demonstrated kinds of prevention measures, but most of them are single or multiple preventive interventions and are not based on evidence. Evidence-based protocol initiated by nurses for syndrome management is scientific, effective, targeted, safe and systematic in stroke units [26]. The evidence-based practice model aimed to address the problems existing in current clinical practice by developing care bundle plans based on a thorough literature review and clinical scenarios, which made up for the conventional nursing mode based on experience and improved the effectiveness and safety of nursing practice.

Applying evidence-based practice methods to our project resulted in a decrease in the incidence of SAP. The infection indicators PCT and CRPH in the postimplementation group were significantly lower than those in the preimplementation group. The care bundle included dysphagia management, feeding management, oral hygiene management, position management, airway management and TCM nursing techniques. The above 6 aspects of the nursing interventions worked together to prevent SAP.

After the intervention, the nutrition indicators ALB and oral hygiene were significantly better in the postimplementation group than in the preimplementation group. Early nutritional risk screening, oral care and swallowing rehabilitation training could help patients increase food intake and improve malnutrition. In addition, the significant improvement in aspiration benefited from standard and systematic screening and management. The cost of medical care was lower than before but did not have statistical significance considering complex factors, such as treatment protocols, the severity of stroke and complications. All factors were combined to reduce the length of hospitalization stay.

### Limitations

This was a single-center EBP project in an urban level 1 stroke center, and was limited by small sample size and restricted time for project implementation. Furthermore, some blood markers were not tested on the same day that the disease attacked, so bias may exist. Finally, the nurses’ compliance with using the entire care bundle needs to be improved in the future. The stroke center was a teaching hospital that had a frequent turnover of clinical nursing staff because of internship rotations, which required attention to who had been trained and who may have missed meetings or training sessions. Nonetheless, the clinically important findings in the prevention of SAP were promising and warrant further investigation with a larger sample.

### Implications for nursing practice

Given the success of the initial project, the practice changes can be expanded to other neurology units in the clinical facility. This is in line with the guidance provided by the Iowa Model of Evidence-Based Practice for care quality improvement [27]. According to this model, the management plan and flow of SAP prevention can be promoted to other units of the neurology department in the future. Additionally, the implementation of evidence-based practice programs requires ongoing education for staff and changes in culture and behaviors. Future research should examine barriers to implementation and adherence to changes when changes are made.

## Conclusion

SAP has presented challenges to nurses who care for patients with acute ischaemic stroke for a long time. An evidence-based practice project to empower nurses to reduce the incidence of SAP was successfully implemented. The results of the project reflect positively on the capacity to implement evidence-based practice in an acute care setting of stroke. These outcomes support the call to question traditional practice, which is necessary to advance the nursing profession in support of improved patient outcomes.

## Supplementary Information

**Additional file 1.**

**Additional file 2.**

**Additional file 3.**

## Abbreviations

A^2^DS^2^: Age, Atrial fibrillation, Dysphagia, Sex, Stroke Severity
NICE: National Institute for Clinical Excellence
NGC: National Guideline Clearinghouse
SING: Scottish Intercollegiate Guidelines Network
CT: Computerized Tomography
MRI: Magnetic Resonance Imaging
SAP: Stroke-associated pneumonia
TCM: Traditional Chinese Medicine
NIHSS: National Institutes of Health Stroke Scale
NRS-2002: Nutrition risk screening-2002
ALB: Serum albumin
WBC: White blood cell
PCT: Procalcitonin
CRPH: Hypersensitive C-reactive protein
