# Supplementary material for: Reducing the incidence of stroke-associated pneumonia: an evidence-based practice
Source: BMC Neurol. 2022 Aug 11;22:297. doi: 10.1186/s12883-022-02826-8 (PMC9367053; doi:10.1186/s12883-022-02826-8)
Supplement: Supplementary file 1 — Additional file 1. [file 12883_2022_2826_MOESM1_ESM.docx]

Supplement 1

The management details of the main points of the evidence-based care bundle

| **Main points** | **Evidence-Based Interventions** | **References** |
| --- | --- | --- |
| **Dysphagia management** | Swallowing screening within 6-hour of admission through EAT-10, 30 ml water swallowing test and VVST.  Swallowing screening is repeated daily for a minimum of one week after initial assessment. | [1-6] |
|  | **Dysphagia rehabilitation**  **Indirect swallowing training** is conducted when patients are conscious, the vital signs are stable, without serious cardiopulmonary complications, breathe steadily, no signs of nausea and vomiting, and can follow instructions.  Training is arranged after meals or half an hour before meals.  The training includes: exercises for muscles around the mouth; neck relaxation; oral sensory training (include ice cotton swab stimulation, K point stimulation, etc.); phonetic training; breathing training; cough training; Mendelsohn technique; and breath-holding swallowing exercises.  **Direct swallowing training (eating through mouth)** is conducted when patients are conscious, the vital signs are stable, without serious cardiopulmonary complications, breath steadily, can produce swallowing reflex, and a small amount of accidental swallowing can be coughed out.  After determining the volume and viscosity of bolus, type of food, and posture and method of feeding, the difficulty of direct training is gradually increased.  While training, some problems occurred during a certain period of ingestion and swallowing were evaluated and recorded to adapt training plans. | [1, 4-5, 7-9] |
| **Feeding modification** | Nutrition assessment is performed with NRS-2002 within 24-hour of admission.  Nutrition screening is repeated weekly after initial assessment. | [1, 4-5, 7] |
|  | **Through mouth**  Adjust the volume and viscosity of bolus based on the assessment of VVST to reduce the risk of aspiration and choking. For example, thickener can be added to the patients' liquid diet to increase the viscosity to form jellylike or pasty boluses, granular food can be taken with medical jelly. Patients start with small volumes (jelly <7 ml, paste <5 ml, granular < 2 ml) and drink a little water (< 2 ml) after each meal. | [1, 4, 7] |
|  | **Through tube**  Tube feeding should be initiated for patients who cannot swallow safely, and nutritional supplements should be considered in patients with malnutrition or identified as high risk of malnutrition.  Check the location of feeding tube before feeding.  In patients with pyloric obstruction, gastroparesis, oesophageal reflux, or aspiration, retropyloric catheterization is used for feeding. | [1-2, 4, 7] |
| **Oral care** | Oral hygiene assessment within 6-hours of admission | [1, 7] |
|  | **Basic oral care**:  Patients with results of grade Ⅲ or above in 30 ml water swallowing test were given special oral care (brushing with negative pressure suction) by nursing staff three times a day.  Patients with results of grade Ⅰ or Ⅱ in 30 ml water swallowing test gargle by themselves before and after meals.  According to the saliva secretion cycle and the eating times, increase the frequency of oral care to remove secretions and food debris. | [1-2, 4, 7] |
| **Airway management** | Teach or assist patients to cough effectively.  Help patients body-turning and backslapping every 2 hours.  Give timely suction when patients cannot automatically discharge sputum. | [2] |
| **Position management** | Patients take food in a sitting or semireclining position, and the head of the bed was raised more than 30 degrees when tube feeding. | [4, 7] |
| **The nursing techniques of TCM** | Acupoint massage twice a day for 5 to 10 minutes per session. The acupoints selection include Baihui, Dazhui, Yifeng, Lianquan and Tiantu.  Chinese medicine enema and abdominal massage for patients with constipation. | [10-14] |
| **Management of caregivers** | Distribute care brochures and organize thematic health education. | [2] |

SAP, Stroke-associated pneumonia; EAT-10, EAT-10 Swallowing Screening Scale; VVST, Volume viscosity swallowing test; NRS-2002, Nutrition risk screening 2002; WBC, White blood cell; PCT, procalcitonin; CRPH, Hypersensitive C-reactive protein; TCM, Traditional Chinese Medicine.

**References**

1. Boulanger JM, Lindsay MP, Gubitz G, Smith EE, Stotts G, Foley N, et al. Canadian Stroke Best Practice Recommendations for Acute Stroke Management: Prehospital, Emergency Department, and Acute Inpatient Stroke Care, 6th Edition, Update 2018. Int J Stroke. 2018;13(9): 949-984. <https://doi.org/10.1177/1747493018786616>
2. Powers WJ, Rabinstein AA, Ackerson T, et al. Guidelines for the Early Management of Patients With Acute Ischemic Stroke: 2019 Update to the 2018 Guidelines for the Early Management of Acute Ischemic Stroke: A Guideline for Healthcare Professionals From the American Heart Association/American Stroke Association. Stroke. 2019; 50(12):e344-e418. https://doi.org/10.1161/STR.0000000000000211.
3. Gittler M, Davis AM. Guidelines for Adult Stroke Rehabilitation and Recovery. JAMA. 2018; 319(8):820-821. doi: 10.1001/jama.2017.22036.
4. Iris C, Catherine D, Jane C, David C, Francesca C, Ali E, et al. Management of patients with stroke identification and management of dysphagia. Scottish Intercollegiate Guidelines Network, 2005.
5. Neurology Society of Chinese Medical Association. Chinese Guidelines for Early Rehabilitation Treatment for Patients with Stroke. Chin J Neurol. 2017; 50(6):405-412. (in Chinese)
6. Moola S. Post-Stroke Dysphagia: Risk Assessment. Joanna Briggs Institute EBP. 2017; 1(29).
7. Chinese Expert Consensus Group on Rehabilitation Assessment and Treatment of Dysphagia. 2017 Expert Consensus on Assessment and Treatment of Dysphagia in China, Part Ⅱ: Treatment and Rehabilitation Management. Chin J Phys Med Rehabil. 2018; 40(1),1-10. (in Chinese) https://doi.org/10.3760/cma.j.issn.0254-1424.2018.01.001
8. Pamaiahgari P. Post-Stroke Dysphasia: oral Motor Exercise. Joanna Briggs Institute EBP. 2018; 8(8).
9. Lizarondo L. Post-Stroke Dysphagia:Nursing Management, Joanna Briggs Institute EBP. 2017; 6(28).
10. Wang XZ, Wang SL. The Clinical Research of Relaxing Therapy’s Prevention on Stroke Associated Pneumonia. Chin J Chin Med. 2010; 25(4): 630-632. (in Chinese)
11. Wu J. Effect of acupoint massage on prevention of stroke-associated pneumonia. J Clini Nurs. 2011; 10(4): 41-42. (in Chinese)
12. Wu SM, Chen DD, Lin SN, Wang Q, Lin HL. Prevention effect on pulmonary infections of patients with dysphagia after stroke. Chin J Nosocomiol. 2015; 25(20): 4706-4708. (in Chinese)
13. Zhang XP, Peng XT, Mo MM, Liu ZY, Wei L. Effects of traditional Chinese medicine Tongfu therapy in prevention of stroke-associated pneumonia: a eta-analysis. Nurs Integr Tradit Chin Western Med. 2019; 5(05): 26-31. (in Chinese)
14. Cai JY, Chen HP. Prevention of Tongfu Method in High-risk Patients with Stroke-Associated Pneumonia. Chin J Tradit Med Sci Technol. 2018; 25(5): 720-722. (in Chinese)
