# Supplementary material for: Reducing the incidence of stroke-associated pneumonia: an evidence-based practice
Source: BMC Neurol. 2022 Aug 11;22:297. doi: 10.1186/s12883-022-02826-8 (PMC9367053; doi:10.1186/s12883-022-02826-8)
Supplement: Supplementary file 2 — Additional file 2. [file 12883_2022_2826_MOESM2_ESM.docx]

Supplement 2

The diagnosis of stroke associated pnuemonia (SAP)

| **Radiology** | **Signs/Symptoms** |
| --- | --- |
| Two or more serial chest radiographs with at least 1 of the following:   1. New or progressive and persistent infiltrate 2. Consolidation 3. Cavitation   NOTE: In patients without underlying pulmonary or cardiac disease (eg, respiratory distress syndrome, bronchopulmonary dysplasia, pulmonary edema, or chronic obstructive pulmonary disease), definitive chest radiograph is acceptable. | For any patient, at least 1 of the following:   1. Fever (>38.8℃ or <100.4°F) with no other recognized cause 2. Leukopenia (<4000 WBC/mm^3^ ) or leukocytosis (≥12,000 WBC/mm^3^ ) 3. For adults ≥ 70 years old, altered mental status with no other recognized cause   and  at least 2 of the following:   1. New onset of purulent sputum or change in character of sputum or increased respiratory secretions or increased suctioning requirements 2. New onset or worsening cough, or dyspnea, or tachypnea 3. Rales or bronchial breath sounds 4. Worsening gas exchange (eg, O_2_ desaturations [eg, PaO_2_/FiO_2_ ≤ 240], increased oxygen requirements, or increased ventilator demand) |

SAP, Stroke-associated pneumonia; WBC, White Blood Cell.
