# Supplementary material for: Reducing the incidence of stroke-associated pneumonia: an evidence-based practice
Source: BMC Neurol. 2022 Aug 11;22:297. doi: 10.1186/s12883-022-02826-8 (PMC9367053; doi:10.1186/s12883-022-02826-8)
Supplement: Supplementary file 3 — Additional file 3. [file 12883_2022_2826_MOESM3_ESM.docx]

Supplement 3

The diagnosis of aspiration

| **Signs/Symptoms** |
| --- |
| For any patient, at least 1 of the following:   1. During the implementation of enteral nutrition, there were obvious choking, wheezing, accelerated respiratory or heart rate, residual enteral nutrient solution in mouth and nose, and nutrient solution residue in sputum after sputum aspiration. 2. The respiratory secretions from subglottic aspiration were detected with pH paper three times a day. When the pH value was less than 7, aspiration was considered. 3. Fiberoptic bronchoscopy revealed gastric contents in the respiratory tract. |

Aspiration was defined as the entry of gastric or oropharyngeal fluids into the lower airways, which may contain bacteria and/or be of low pH, or exogenous substances (eg, ingested food particles or liquids, fresh water, etc.) .
